# Phenotypic and genotypic characterization of azacitidine-sensitive and resistant SKM1 myeloid cell lines

**DOI:** 10.18632/oncotarget.2024

**Published:** 2014-05-27

**Authors:** Thomas Cluzeau, Alix Dubois, Arnaud Jacquel, Frederic Luciano, Aline Renneville, Claude Preudhomme, Jean Michel Karsenti, Nicolas Mounier, Pierre Rohrlich, Sophie Raynaud, Bernard Mari, Guillaume Robert, Patrick Auberger

**Affiliations:** ^1^ INSERM U1065, Mediterranean Center for Molecular Medicine (C3M), Nice, France; ^2^ INSERM U1065, Team 2: Cell Death, Differentiation, Inflammation and Cancer; ^3^ Equipe labellisée par la Ligue Nationale Contre le Cancer (2011-2013), Paris, France; ^4^ University of Nice, Nice, France; ^5^ Laboratory of Hematology, Biology and Pathology Center, CHRU of Lille, Lille, France; ^6^ Department of Clinical hematology and Transplantation, CHU of Nice, Nice, France; ^7^ Oncohematology laboratory, CHU of Nice, Nice, France; ^8^ Molecular and Cellular Pharmacology Institute, CNRS, Sophia-Antipolis, France

**Keywords:** MDS, AML, Azacitidine, Polyploidy, TET2, ASLX1

## Abstract

In the present study, we provide a comparative phenotypic and genotypic analysis of azacitidine-sensitive and resistant SKM-1 cell lines. Morphologically, SKM1-R exhibited increase in cell size that accounts for by enhanced ploidy in a majority of cells as shown by cell cycle and karyotype analysis. No specific Single Nucleotide Polymorphism (SNP) alteration was found in SKM1-R cells compared to their SKM1-S counterpart. Comparative pangenomic profiling revealed the up-regulation of a panel of genes involved in cellular movement, cell death and survival and down-regulation of genes required for cell to cell signaling and free radical scavenging in SKM1-R cells. We also searched for mutations frequently associated with myelodysplastic syndromes (MDS) and found that both cell lines harbored mutations in TET2, ASLX1 and TP53. Collectively, our data show that despite their different morphological and phenotypic features, SKM1-S and SKM1-R cells exhibited similar genotypic characteristics. Finally, pangenomic profiling identifies new potential pathways to be targeted to circumvent AZA-resistance. In conclusion, SKM1-R cells represent a valuable tool for the validation of new therapeutic intervention in MDS.

## INTRODUCTION

Myelodysplastic syndromes (MDS) are clonal hematopoietic stem cell disorders characterized by ineffective hematopoiesis leading to blood cytopenias, especially anemia, and often evolving to acute myeloid leukemia (AML) [1]. MDS largely predominate in the elderly [2-4]. They are classified based on morphology and blast cell percentage in the blood and the bone marrow [5]. Main prognostic factors of MDS, for progression to AML and survival, include the number and importance of the cytopenia, the percentage of marrow blasts and finally bone marrow cytogenetic abnormalities [6]. Those factors are combined in an International Prognostic Scoring System that distinguishes 4 subgroups with different risk of progression to AML and survival (low, intermediate 1 (int-1), intermediate 2 (int-2) and high). Low and int-1 subgroups are often considered together as favorable or low risk MDS, int-2 and high subgroups are classified as unfavorable or high risk MDS (HR-MDS). IPSS scoring has been recently revised allowing better stratification of MDS patients [7].

Aracytine-based chemotherapy yields complete remission (CR) rates of 50% in HR-MDS [8], but is limited to a minority of MDS patients, whose age and general condition do not preclude such treatment. In addition, duration of CR is generally short (about 1 year) and most patients relapse. Lower intensity chemotherapy regimens, mainly low dose aracytine have been largely used in elderly MDS patients, with CR and partial remission (PR) rates of only 20% [9-11]. In addition, these chemotherapy regimens have poor efficacy in patients with an unfavourable karyotype [12].

Several consistent studies demonstrated that increased and aberrant gene hyper-methylation occur during progression from low to high-risk MDS [13]. Based on the hypothesis that hyper-methylation might favour leukemogenesis by silencing tumor suppressor genes, demethylating agents including AZA have been introduced for the treatment of MDS with the aim to antagonize this process [14, 15]. Two phase-III studies demonstrated a survival benefit of AZA over conventional care regimen [16, 17]. Nevertheless, only 50 to 60% of the patients respond to AZA and most of responders relapse within 12 to 15 months. In relapse or refractory patients, median survival is around 6 months [18]. Therefore new treatments are urgently needed for these patients. In this line, several drugs have been tested in those patients, with so far no demonstrated effect on survival.

To get insight into the mechanisms of AZA resistance, we generated AZA-resistant SKM-1 cells from the parental SKM-1 MDS/AML cell line, initially isolated from a patient with a refractory anemia with an excess of blasts (RAEB-2) in transformation [19]. We described herein, the phenotypic and genotypic features of both SKM1-S and SKM1-R cells. We found that SKM1-R cells exhibit different morphological and phenotypic features as compared to SKM1-S cells, with no global genotypic alterations. Finally, pangenomic profiling of both cell lines shows preferential modulation of genes involved in cell migration, death and survival.

## RESULTS AND DISCUSSION

**Figure 1:**
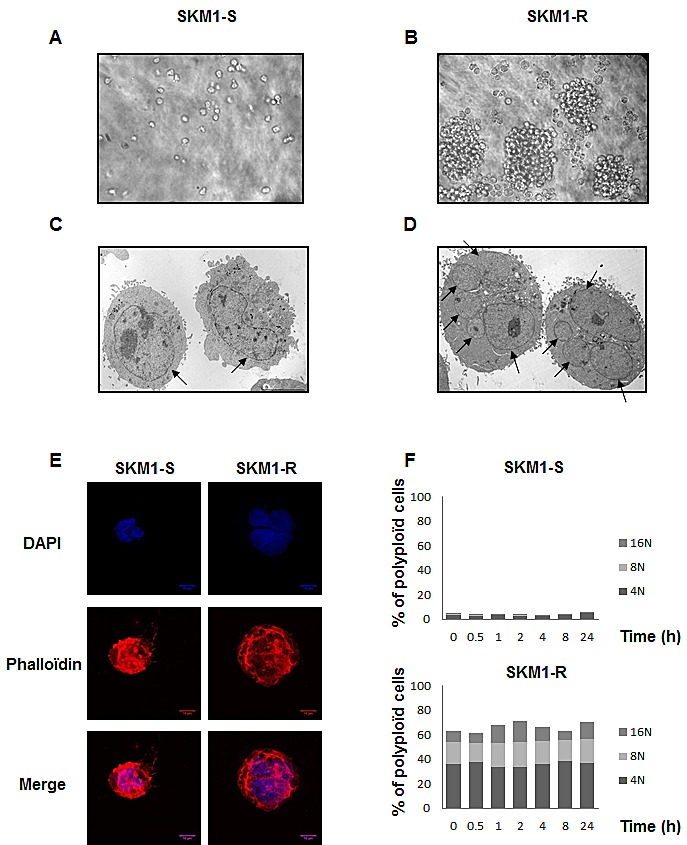
Morphologic features of SKM1-S and SKM1-R cells: Panels A and B: Phase contrast analysis of SKM1-S and SKM1-R cells (X100) Panels C and D: Electron microscopy images of SKM1-S and SKM1-R cells at different magnification (X4000). Panel E: SKM1-S and SKM1-R cells were co-stained with DAPI (blue) and phalloïdine and analyzed by confocal microscopy (red). Panel F: Quantification of ploïdy in SKM1-S and SKM1-R cells: SKM1 cells were washed, fixed in ethanol 70%, and finally left over night at −20°C. The cells were next incubated in PBS, containing 3μg/ml RNase A and 40μg/ml of propidium iodide (PI) for 30min at 4°C. Cell distribution across the different phases of the cell cycle and/or DNA content was analyzed with a Miltenyi cytometer. Histograms represent the percentage of cells with DNA content of 2N, 4N, 8N and 16N

**Figure 2:**
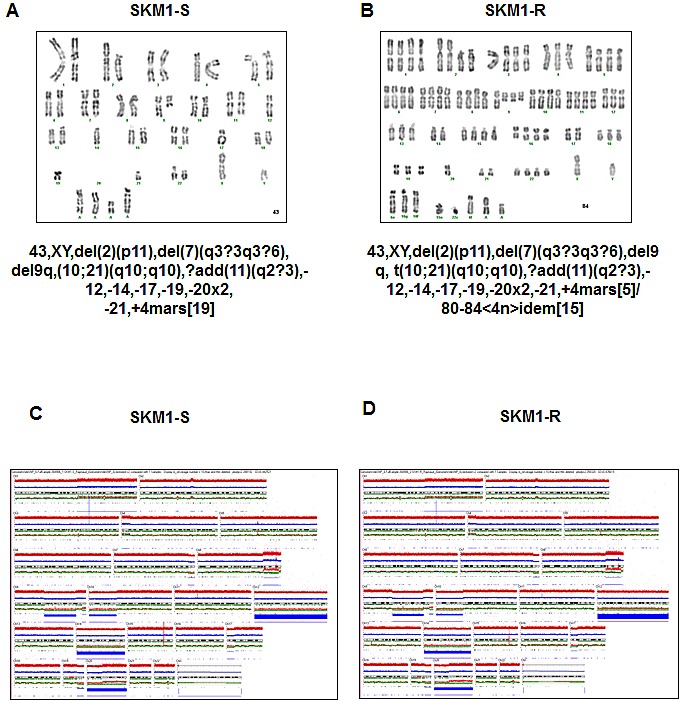
Cytogenetic characteristics of SKM1-S and SKM1-R cells Panels A and B: Conventional karyotype of SKM1-S and SKM1-R cell lines. Panels C and D: Molecular karyotype of the SKM1-S and SKM1-R cell lines in SNP array.

Phase contrast images of SKM1-S and SKM1-R cell lines revealed that conversely to SKM1-S cells that grew individually, SKM1-R cells formed clones containing several tens of cells (Figures 1A and). Fluorescence microscopy experiments confirmed increased size and ploidy in SKM1-R cells stained with both DAPI (blue) and phalloïdine to visualize the actin network (red) (Figure 1E). Electron microscope images also showed that SKM1-R cells exhibited increased size and ploïdy (Figure 1F). As shown on Figure 1 C and D, most of cells exhibited micronuclei, with some cells containing up to eight of these structures (arrows). Finally, quantitative flow cytometry analysis of DNA content indicated that less than 5% of SKM1-S cells were tetraploïd whereas up to 40% of SKM1-R cells exhibited tetraploidy. Importantly, around 20% of SKM1-R cells had an 8N DNA content and 8% had a 16N DNA content (Figure 1F). The same distribution of DNA content was maintained during 96h of culture. In conclusion, SKM1-R cells were found to be near tetraploid as compared to their SKM1-S counterpart, that were hyperdiploid. Besides ploidy, the global caryotype assessed by conventional cytogenetic (Figures 2A and 2B) or SNP array analysis of both cell clones (Figures 2C and 2D) was strictly identical between SKM1-S and SKM1-R cells. Indeed, no evidence of cytogenetic abnormalities and no SNP alterations were found in SKM1-R cells compared to their SKM1-S counterpart. Most of the time, tetraploidy results from the endoduplication of an previously haploid karyotype. This could be indeed the case here, since no other chromosomal abnormalities arose in the SKM1-S versus SKM1-R clone. The reasons underlying tetraploidy in SKM1-R cell is currently unknown. We have shown previously that foretinib, a Polo Like Kinase 1 (PLK1) inhibitor induced polyploidy in an imatinib-resistant K562 cell line via a caspase-2-dependent mechanism [20]. However, PLK1 was found to be expressed at identical levels in SKM1-S and SKM1-R cell lines (results not shown). Although SKM1-R cells have been described previously to exhibit impaired mitochondrial apoptosis in response to AZA[21], caspase-2 expression and activity were not affected in these cells (data not shown). It would be of interest to determine whether AZA triggers caspase-2 activation in SKM1-S and SKM1-R cells and whether foretinib is able to restore cell death in SKM1-R cells.

In addition, long-term incubation of SKM1-S cells with different concentrations of AZA (up to 96h) failed to reproduce tetraploidy suggesting that polyploidy is a feature of AZA-resistant cells but is not a direct consequence of AZA treatment. Interestingly, increased ploidy has been already reported in a multiple myeloma cell line resistant to the proteasome inhibitor bortezomib [22]. In this line, the observation that the SKM1-R cell line is nearly tetraploid could be physiologically relevant. Indeed, it has been reported earlier that the plasma cells of patients who developed bortezomib resistance evolved from hyperdiploidy to tetraploidy [23]. The relationship between tetraploidy and AZA resistance is not clear at present but one could speculate that modulation of gene expression in SKM1-R cells, such as increased cyclin D1 expression that normally lead to mitotic arrest and apoptosis in SKM1-S could be overcome in some SKM1-R cells leading to impaired apoptosis, survival, amplification and abnormal cytokinesis. It has been also reported that tetraploidy is a rare event and a poor pronostic factor in MDS [24]. In line with these findings, it would be interesting to compare if tetraploidy is more frequently observed in primary bone marrow cells from AZA-resistant MDS and AML patients.

A significant advance in the pathogenesis of myeloid malignancies is the recent discovery of mutations in gene encoding enzymes involved in the regulation of DNA conformation [25-27]. We thus sequenced the 14 most frequently mutated genes in MDS to characterize molecularly our cell lines and to investigate whether long-term treatment of SKM1-R cells with AZA may impact on the frequency of such mutations (for the primer sequences, please refer to sup Table 1). As shown on Table 1, mutations in TP53, TET2 and ASLX1 were detected in both SKM1-S and SKM1-R cells, whereas expression of the other genes tested was not affected. We concluded that AZA does not modify the status of mutations of SKM1 cells. Interestingly, mutations of both TET2 and ASLX1 were found in SKM-1 cells, as it is the case in a significant proportion of MDS and AML patients. Therefore our SKM1-R cell line represents an excellent cell line model, to investigate the mechanisms of AZA resistance but to decipher the functional roles of TET2 and ASXL1 in the context of MDS.

**Table 1:** Analysis of the most frequently MDS-associated gene mutations in SKM1-S and SKM1-R cells

	SKMl-S	SKMl-R
TPS3	c.743G>A,p. R248Q(HO)	c.743G>A,p. R248Q(HO)
RUNXl	WT	WT
NRAS	WT	WT
KRAS	WT	WT
TET2	c. 4253_4254insTT, p.l419CfsX30(HE)	c.4253 4254insTT, p.1419CfsX30(HE)
ASXLl	c.1773C>A,p.YS91X (HO)	c.1773C>A, p.YS91X (HO)
CBL	WT	WT
EZH2	WT	WT
IDH1	WT	WT
IDH2	WT	WT
DNMT3A	WT	WT
SF3Bl	WT	WT
U2AF35	WT	WT
SRSF2	WT	WT
ZRSR2	WT	WT

**Figure 3:**
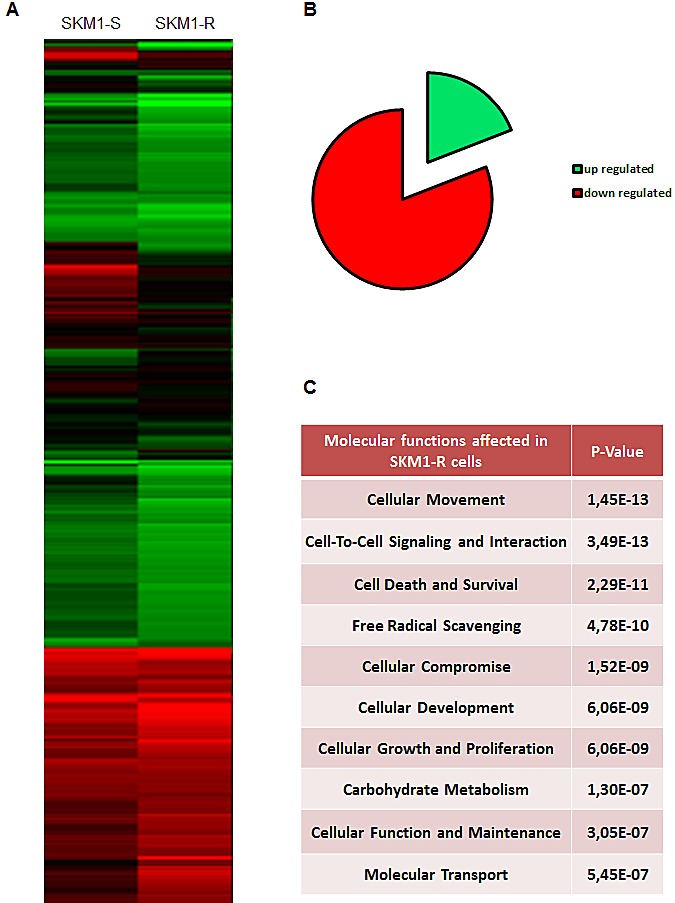
Pangenomic analysis of SKM1-S and SKM1-R cells Panel A: Hierarchical clustering of samples using the best genes discriminating the SKM1-S and SKM1-R cells RNA samples from SKM1-S and SKM1-R cells (in basal condition) were harvested and expression profiles were determined using pangenomic arrays. Gene expression patterns were determined by the 2-way hierarchical clustering method using the 422 genes selected for discriminating the SKM1-S and SKM1-R cells. Each square represents the expression level of a given gene and sample, colors vary from green to red for the lowest to the highest gene expression levels, respectively. The distance corresponds to a Manhattan distance. Panel B shows the 10 most affected molecular functions in SKM1-R cell. Panel C depicts the repartition of the 89 most regulated genes in SKM1-R versus SKM1-S cells.

Finally, to identify genes potentially involved in the resistance of SKM1-R cells to AZA, we performed microarray analysis of both SKM1-S and SKM1-R cells (Figure 3A). Pangenomic profiling revealed up-regulation of a panel of genes involved in cellular movement, cell death and survival, cellular compromise, cellular growth and proliferation (Figure 3B). Our micro-array analysis also evidenced down-regulation of genes required for cell to cell signaling and interaction, free radical scavenging and cellular development in SKM1-R cells (Figure 3C). Finally, supplemental Table 2 shows the list of the best 89 genes discriminating WT from AZA-resistant SKM1 cells. Among them, 80% were down regulated and 20% up regulated. This different pattern of gene expression could be useful to identify and characterize new potential targetable pathways in MDS and to validate new therapeutic strategies for myeloid malignancies.

## MATERIALS AND METHODS

### Electronic microscopy

The cells were fixed with 2% formaldehyde, in 0.1M Na cacodylate, pH 7.4 for 1 h at 41°C. The pellets were rinsed in cacodylate buffer, post-fixed in 1% OsO4 for 1 h, dehydrated through graded alcohols, and embedded in epoxy resin. Oriented 1mm sections were obtained with diamond knives and multiple areas were thin sectioned, mounted on copper mesh grids, and stained with uranyl acetate and lead citrate. Ultrathin sections were examined on a Jeol 1200 XII electron microscope.

### Confocal microscopy

The cells prepared for fluorescence staining were grown on glass coverslips. They were washed with ice-cold PBS and were successively fixed and permeabilized with 1% PFA and PBS plus 0.1% Triton X-100, respectively. Then, the cells were incubated for 1 h with phalloidin. Finally, the cells were incubated with 1 mg/ml DAPI, mounted on glass slides in Fluoromount-G (Southern Biotechnology Associates, 0100-01) and photographed with a confocal laser microscope (Carl Zeiss, LSM-510-Meta).

### Ploidy and cell cycle analysis

After treatment, the cells were washed, fixed in ethanol 70% and, finally, left overnight at -20°C. Cells were next incubated in PBS, 3 μg/ml RNase A and 40 μg/ml of propidium iodide (PI) for 30 min at 4°C. Cell distribution across the different phases of the cell cycle or DNA content was analyzed with a Miltenyi cytometer [20].

### Conventional cytogenetic

Cytogenetic analyses were performed on metaphase cells derived from 24-h unstimulated SKM1-S and SKM1-R cell lines. Complete karyotyping was done with a minimum of 20 metaphases analyzed. Briefly, the cells were synchronized using fluorodeoxyuridine, uridine and thymidine. Colcemid was added to culture (0.05 g/ml) for half an hour. After incubation in hypotonic solution for 30 min (0.075 MKCL), cells were fixed with Carnoby's solution (3 parts methanol to 1 part glacial acetic acid). After G-banding, karyotypes were interpreted according to the 2009 International System for Human Cytogenetic Nomenclature.

### SNP array

We performed genome wide single nucleotide polymorphism (SNP) analysis using SNP 6.0 arrays (Affymetrix, High Wycombe, U.K) on SKM1-S and SKM1-R cell lines. DNA was prepared for hybridization according to the manufacturers' recommendations. Affymetrix CEL files for each sample were analyzed using the Genotyping Console software (v3.0.2). Genotyping was performed using Birdseed V2 algorithm. Unpaired Copy Number and LOH analysis was performed with Regional GC correction. Copy number and UPD were also analyzed using the Copy Number Analyzer for GeneChip (CNAG version 3.3.0.1) algorithm (http://www.genome.umin.jp/CNAGtop2.html) [28].

### Gene expression analyses

Microarray analyses were performed on the GeneChip Human Gene 1.0 ST Array (Affymetrix, Santa Clara, CA 95051, USA), according to the manufacturer's instructions. Microarray data are archived in GEO, and on the MEDIANTE database developed by the laboratory (Lebrigand and Barbry, bioinformatics 2011). Normalization was performed using RMA and log2 ratio was calculated for 1 different contrast: WT NT versus AZA-R NT. The best discriminating genes were selected using the following threshold: log2 (intensity) >7 in at least one experimental condition and log2 (ratio) >2 for at least one contrast. Hierarchical clustering was done with the Multi-Experiment Viewer (MeV) program, using a Manhattan distance metric and average linkage. Biological theme analysis was performed using Ingenuity Pathway Analysis software™ (IPA™).

### Gene mutations analysis

*TP53* mutation screening was performed using Sanger sequencing. Exons 5 to 8 of *TP53* were amplified from genomic DNA using the intronic primers indicated in Supplemental Table 1. The purified PCR products were sequenced in both directions using the BigDye^®^ Terminator Cycle Sequencing Kit (Applied Bio-systems, Foster City, CA) and analyzed on the Applied Biosystems 3730xl Genetic Analyzer. The Seqscape software version 2.7 (Applied Biosystems) was used to detect sequence variations. Mutations in *ASXL1* (exon 12), *CBL* (exons 8-9), *DNMT3A* (exons 8-9 and 11-23), *IDH1*R132, *IDH2*R140, *IDH2*R172, *N-/K-RAS* (exons 2-3), *RUNX1* (exons 3-8), and *TET2* (exons 3-11) were screened by Sanger sequencing, as previously described [29-36].

## SUPPLEMENTARY TABLES


