# Supplementary material for: Phenotypic and genotypic characterization of azacitidine-sensitive and resistant SKM1 myeloid cell lines
Source: Oncotarget. 2014 May 27;5(12):4384–91. doi: 10.18632/oncotarget.2024 (PMC4147331; doi:10.18632/oncotarget.2024)
Supplement: Supplementary file 1 [file oncotarget-05-4384-s001.pdf]

Phenotypic and Genotypic Characterization of Azacitidine-Sensitive and Resistant SKM1 Myeloid Cell Lines

Supplementary Material

Supplemental Table 1: Sequences of the primers used for mutational analysis of MDS cell lines

	Forward primer	Reverse primer
	**SRSF2**	
**SRSF2_ex1**	GGCCGCCACTCAGAGCTA	ACCTCACAAAGGTCCGCG
	**ZRSR2**	
**ZRSR2_ex1-2**	TCTCGACTCTTAGGCCCGCCCTTT	CCTTCTGACACTGGGGCTTCAAG
**ZRSR2_ex3**	GCTTGTGTTGTACCAAAGAAGG	ACAGAAGACTGGTACTGGTTAG
**ZRSR2_ex4-5**	TTTGCTCTCGTGTGTGTGTG	CCCAAACTCTGACATGCCTA
**ZRSR2_ex6**	TGTTCCACTTGAGATTCTTAACCA	GATCTAACTAACCTACCACG
**ZRSR2_ex7**	CTTGATTGCCTGTTCCAACT	GTCTGATGACGGACATTTGA
**ZRSR2_ex8**	ATGCCTGGTCTAAAGCAGTT	TTATAGAGTGCTAGCGTGCC
**ZRSR2_ex9**	GCAAGAGTCAGCTAGTATCT	TCCAGTGGAAAAATCCCAGA
**ZRSR2_ex10**	GAACTTGGTGGTCCTACAAT	TCCCCCAAAGAATATCCCTT
**ZRSR2_ex11**	AGTGCTGTTTCATCACTGTGC	AACCCATCTGCGTTCATAGC
	**U2AF1**	
**U2AF1_ex2**	GCTGCTGACATATTCCATGTG	TCTCAGACCTTCCACTGGAAGT
**U2AF1_ex6**	AAAGTCTTATTAAAGCGTGGATGG	CGAACTGTGCTCAGTCACGTC
	**SF3B1**	
**SF3B1_ex13-14**	TGATGTGAAAGTGTAGCTTC	GGCAACATAGTAAGACCCTGT
**SF3B1_ex15-16**	TGTTGGGGCATAGTTAAAACCT	TGTTAGAACCATGAAACATATCCA
	**KRAS**	
**KRAS_ex2**	AAAGGTACTGGTGGAGTATTTGA	CATGAAAATGGTCAGAGAAACC
**KRAS_ex3**	CAGACTGTGTTTCTCCCTTC	TAAACCCACCTATAATGGTG
	**NRAS**	
**NRAS_ex2**	GGCCGATATTAATCCGGTGT	TGGGTAAAGATGATCCGACA
**NRAS_ex3**	CAAGTGGTTATAGATGGTGAAAC	CAAATGACTTGCTATTATTGATG
	**CBL**	
**CBL_ex8**	GGACCCAGACTAGATGCTTTCT	GAAAATACATTTCCTAGAGATCAAAA A
**CBL_ex9**	CTGGCTTTTGGGGTTAGGTT	TCGTTAAGTGTTTTACGGCTTT
	**RUNX1**	
**RUNX1_ex3**	AGCTGCTTGCTGAAGATCCG	GCCTGTCCTCCCACCACCCTCTC
**RUNX1_ex4**	CATCCCTGATGTCTGCATTTGTCC	GTGGGTTTGTTGCCATGAAACG
**RUNX1_ex5**	TGTTCAGGCCACCAACCTCATTC	CCCAAGGAATCTGAGACATGGTCC
**RUNX1_ex6**	CCTGCTCCCCACAATAG	GGTGCAGGAGAGGCGGGCAG
**RUNX1_ex7**	AATCCCACCCCACTTTACAT	CTCAGCTGCAAAGAATGTGT
**RUNX1_ex8**	TCCGTTCTCTTGCCCGC	GGCCTGGCGCCTCAGTA
	**EZH2**	
**EZH2_ex2**	GGTGATCATATTCAGGCTGG	AAACTTATTGAACTTAGGAGGGG
**EZH2_ex3**	TTTCTCCTTTCCTCTCCTTCA	TCCAATAGCATAAACCAAAAGATG
**EZH2_ex4**	GGCTACAGCTTAAGGTTGTCCT	CTGTCTTGATTCACCTTGACAAT
**EZH2_ex5**	AAATCTGGAGAACTGGGTAAAGAC	TCATGCCCTATATGCTTCATAAAC
**EZH2_ex6**	AGGCTATGCCTGTTTTGTCC	AAAAGAGAAAGAAGAAACTAAGCCC
**EZH2_ex7**	CTGACTGGCATTCCACAGAC	AAGTGTAGTGGCTCATCCGC
**EZH2_ex8**	CATCAAAAGTAACACATGGAAACC	TTGTAATAAATGATAGCACTCTCCAA G
**EZH2_ex9**	TCCATTAATTGACTTTTCCAGTG	ACCTCCACCAAAGTGCAAAG
**EZH2_ex10**	TTCTCTTCCATCAAAATGAGTTTTAG	TCCTCACAACACGAACTTTCAC

**EZH2_ex11**	GAGTTGTCCTCATCTTTTCGC	CCAAGAATTTTCTTTGTTTGGAC
**EZH2_ex12**	AAGAATGGTTTGCCTAAATAAGAC	CCTTGCCTGCAGTGTCTATC
**EZH2_ex13**	TCTTGGCTTTAACGCATTCC	CAAATTGGTTTAACATACAGAAGGC
**EZH2_ex14**	TGATCGTTTCCATCTCCCTG	AGGGAGTGCTCCCATGTTC
**EZH2_ex15**	GAGAGTCAGTGAGATGCCCAG	TTTGCCCCAGCTAAATCATC
	**Forward primer**	**Reverse primer**
**EZH2_ex16**	TTTTTGATGATGTGATTGTGTTTT	TGGCAATTCATTTCCAATCA
**EZH2_ex17**	TTCTGTCAGGCTTGATCACC	CTCGTTTCTGAACACTCGGC
**EZH2_ex18**	AGGCAAACCCTGAAGAACTG	TTCCAATTCTCACGTCAAAGGTA
**EZH2_ex19**	CCGTCTTCATGCTCACTGAC	AAAAACCCTCCTTTGTCCAGA
**EZH2_ex20**	CTTCAGCAGGCTTTGTTGTG	GGGGAGGAGGTAGCAGATG
**ASXL1**		
**ASXL1_ex12_1**	AGGTCAGATCACCCAGTCAGTT	TAGCCCATCTGTGAGTCCAACTGT
**ASXL1_ex12_2**	AGAGGACCTGCCTTCTCTGAGAAA	TTCGATGGGATGGGTATCCAATGC
**ASXL1_ex12_3**	ACTTGAAAACCAAGGCTCTCGT	GCAACCATCCCATCTGTCCTTGTA
**ASXL1_ex12_4**	GGTGGACAAGGATGAGAAACCCAA	TGTCCTGTGACATAGCACGGACTT
**ASXL1_ex12_5**	TGGATTCCAAAGAGCAGTTCTCTTC	CATGACAAAGGGCATCCCTTCCAA
**ASXL1_ex12_6**	ACAGGAAAGCTACTGGGCATAGTC	CAAGAGTGCTCCTGCCTAAAGAGT
**DNMT3A**		
**DNMT3A_ex23**	TCCTGCTGTGTGGTTAGACG	TTTTTCTCTTCTGGGTGCTGA
**TET2**		
**TET2_fragment1**	TGAACTTCCCACATTAGCTGGT	GAAACTGTAGCACCATTAGGCATT
**TET2_fragment2**	CAAAAGGCTAATGGAGAAAGACGTA	GCAGAAAAGGAATCCTTAGTGAACA
**TET2_fragment3**	GCCAGTAAACTAGCTGCAATGCTAA	TGCCTCATTACGTTTTAGATGGG
**TET2_fragment4**	GACCAATGTCAGAACACCTCAA	TTGATTTTGAATACTGATTTTCACCA
**TET2_fragment5**	TTGCAACATAAGCCTCATAAACAG	ATTGGCCTGTGCATCTGACTAT
**TET2_fragment6**	GCAACTTGCTCAGCAAAGGTACT	TGCTGCCAGACTCAAGATTTAAA
**TET2_fragment7**	ATACTACATATAATACATTCTAATTCCCTCACTG	TGTTTACTGCTTTGTGTGTGAAGG
**TET2_fragment8**	CATTTCTCAGGATGTGGTCATAGAAT	CCCAATTCTCAGGGTCAGATTTA
**TET2_fragment9**	AGACTTTATGTATCTTTCATCTAGCTCTGG	ACTCTCTTCCTTTCAACCAAAGATT
**TET2_fragment10**	ATGCCACAGCTTAATACAGAGTTAGAT	TGTCATATTGTTCACTTCATCTAAGCTAAT
**TET2_fragment11**	GATGCTTTATTTAGTAATAAAGGCACCA	TTCAACAATTAAGAGGAAAAGTTAGAATAATATTT
**TET2_fragment12**	TGTCATTCCATTTTGTTTCTGGATA	AAATTACCCAGTCTTGCATATGTCTT
**TET2_fragment13**	CTGGATCAACTAGGCCACCAAC	CCAAAATTAACAATGTTCATTTTACAATAAGAG
**TET2_fragment14**	GCTCTTATCTTTGCTTAATGGGTGT	TGTACATTTGGTCTAATGGTACAACTG
**TET2_fragment15**	AATGGAAACCTATCAGTGGACAAC	TATATATCTGTTGTAAGGCCCTGTGA
**TET2_fragment16**	CAGAGCTTTCTGGATCCTGACAT	GCCCACGTCCATGAGAACTATACTAC
**TET2_fragment17**	TCTAAGCTCAGTCTACCACCCATCCATACA	TGCTCGCTGTCTGACCAGACCTCATCG
**IDH1**		
**IDH1-R132**	GTGGCACGGTCTTCAGAGA	TTCATACCTTGCTTAATGGGTGT
**IDH2**		
**IDH2-R140**	TGAAAGATGGCGGCTGCAGT	GGGGTGAAGACCATTTTGAA
**IDH2-R172**	AGCCATCATCTGCAAAAC	TGTGGCCTTGTACTGCAGAG
**TP53**		
**TP53_ex5**	AAGCTCCTGAGGTGTAGACGC	GGGCCAGACCTAAGAGCAATC
**TP53_ex6**	CTGCTCAGATAGCGATGGTG	AGGCCCTTAGCCTCTGTAAGC
**TP53_ex7**	CATCCTGGCTAACGGTGAAAC	AGAAATCGGTAAGAGGTGGGC
**TP53_ex8**	GTTGGGAGTAGATGGAGCCTG	TTGGGCAGTGCTAGGAAAGAG

Supplemental Table 2 :List of the best 89 genes discriminating WT from AZA-Resistant (R) cells. Affymetrix Probe Set IDs and NCBI Gene Accessions give access to transcripts. Logarithm (base 2) of the ratio (R / WT) are represented.

Down-Regulated Genes

Gene Symbol	Probe Set ID	Gene Accession	Cytoband	Gene Description	log2 (R /WT)
CLC	8036755	NM_001828	19q13.1	Charcot-Leyden crystal protein	-7,60
CD33	8030804	NM_001772	19q13.3	CD33 molecule	-4,66
BPI	8062444	NM_001725	20q11.23-q12	bactericidal/permeability-increasing protein	-4,30
FXYD5	8027778	NM_144779	19q12-q13.1	FXYD domain containing ion transport regulator 5	-4,01
CCDC92	7967486	NM_025140	12q24.31	coiled-coil domain containing 92	-3,76
IGFBP2	8048205	NM_000597	2q33-q34	insulin-like growth factor binding protein 2, 36kDa	-3,69
LPL	8144917	NM_000237	8p22	lipoprotein lipase	-3,68
S100A8	7920244	NM_002964	1q21	S100 calcium binding protein A8	-3,66
IFITM1	7937335	NM_003641	11p15.5	interferon induced transmembrane protein 1 (9-27)	-3,59
FCN1	8165011	NM_002003	9q34	ficolin (collagen/fibrinogen domain containing) 1	-3,53
HP	7997188	NM_005143	16q22.1	haptoglobin	-3,53
CRABP1	7985159	NM_004378	15q24	cellular retinoic acid binding protein 1	-3,44
AES	8032576	NM_198969	19p13.3	amino-terminal enhancer of split	-3,42
FAM163A	7907769	NM_173509	1q25.2	family with sequence similarity 163, member A	-3,41
CD37	8030277	NM_001774	19q13.3	CD37 molecule	-3,33
PTPN22	7918657	NM_015967	1p13.3-p13.1	protein tyrosine phosphatase, non-receptor type 22 (lymphoid)	-3,25
SLC44A2	8025672	NM_020428	19p13.1	solute carrier family 44, member 2	-3,15
TARP	8139100	M30894	7p15-p14	TCR gamma alternate reading frame protein	-3,12
PEPD	8036010	NM_000285	19q12-q13.2	peptidase D	-3,10
BST2	8035304	NM_004335	19p13.2	bone marrow stromal cell antigen 2	-3,09
ENTPD1	7929511	NM_001776	10q24	ectonucleoside triphosphate diphosphohydrolase 1	-3,05
FPR2	8030860	NM_001462	19q13.3-q13.4	formyl peptide receptor 2	-3,01
OCLN	8105908	NM_002538	5q13.1	occludin	-2,97
NRP2	8047738	NM_201266	2q33.3	neuropilin 2	-2,97
ERRFI1	7912157	NM_018948	1p36	ERBB receptor feedback inhibitor 1	-2,94
FUCA1	7913694	NM_000147	1p34	fucosidase, alpha-L- 1, tissue	-2,92
MGAM	8136662	NM_004668	7q34	maltase-glucoamylase (alpha-glucosidase)	-2,89
GLIPR2	8155192	NM_022343	9p13-p12	GLI pathogenesis-related 2	-2,89
STXBP1	8158059	NM_003165	9q34.1	syntaxin binding protein 1	-2,80
SMPDL3B	7899407	NM_014474	1p35.3	sphingomyelin phosphodiesterase,	-2,67

				acid-like 3B	
RAB13	7963986	NM_002870	1q21.2	RAB13, member RAS oncogene family	-2,61
GNA11	8024557	NM_002067	19p13.3	guanine nucleotide binding protein (G protein), alpha 11 (Gq class) protein (G protein), alpha 11 (Gq class)	-2,58
CABLES1	8020495	NM_138375	18q11.2	Cdk5 and Abl enzyme substrate 1	-2,58
TMEM205	8034210	NM_198536	19p13.2	transmembrane protein 205	-2,53
PRG2	7948221	NM_002728	11q12	proteoglycan 2, bone marrow (natural killer cell activator, eosinophil granule major basic protein)	-2,52
TPCN1	7958960	NM_001143819	12q24.13	two pore segment channel 1	-2,49
ASS1	8158671	NM_000050	9q34.1	argininosuccinate synthetase 1	-2,49
PTCH1	8162533	NM_001083603	9q22.3	patched homolog 1 (Drosophila)	-2,45
LPHN1	8034783	NM_001008701	19p13.2	latrophilin 1	-2,44
MOSC2	7909866	NM_017898	1q41	MOCO sulphurase C-terminal domain containing 2	-2,42
CDA	7898655	NM_001785	1p36.2-p35	cytidine deaminase	-2,41
ADARB1	8069178	NR_027673	21q22.3	adenosine deaminase, RNA-specific, B1 (RED1 homolog rat)	-2,40
CCL4L1	8006621	NM_001001435	17q12	chemokine (C-C motif) ligand 4-like 1	-2,40
TYROBP	8036224	NM_003332	19q13.1	TYRO protein tyrosine kinase binding protein	-2,38
CYP2S1	8028991	NM_030622	19q13.1	cytochrome P450, family 2, subfamily S, polypeptide 1	-2,38
ZNF57	8024532	NM_173480	19p13.3	zinc finger protein 57	-2,36
GALR2	8010030	NM_003857	17q25.3	galanin receptor 2	-2,32
PCSK6	7991602	NM_002570	15q26.3	proprotein convertase subtilisin/kexin type 6	-2,31
CYP4F3	8026456	NM_000896	19p13.2	cytochrome P450, family 4, subfamily F, polypeptide 3	-2,29
CLEC5A	8143471	NM_013252	7q33	C-type lectin domain family 5, member A	-2,26
BCAT2	8038202	NM_001190	19q13	branched chain aminotransferase 2, mitochondrial	-2,24
SBF2	7946516	NM_030962	11p15.4	SET binding factor 2	-2,24
GPR114	7996064	NM_153837	16q21	G protein-coupled receptor 114	-2,23
ICAM3	8033987	NM_002162	19p13.3-p13.2	intercellular adhesion molecule 3	-2,21
CD9	7953291	NM_001769	12p13.3	CD9 molecule	-2,20
CD4	7953428	NM_000616	12pter-p12	CD4 molecule	-2,20
OLR1	7961142	NM_002543	12p13.2-p12.3	oxidized low density lipoprotein (lectin-like) receptor 1	-2,19
PHACTR3	8063739	NM_080672	20q13.32-q13.33	phosphatase and actin regulator 3	-2,17
PTPRU	7899562	NM_133178	1p35.3-p35.1	protein tyrosine phosphatase, receptor type, U	-2,17
ZNF252	8153935	NR_023392	8q24.3	zinc finger protein 252	-2,14
PADI2	7912937	NM_007365	1p36.13	peptidyl arginine deiminase, type II	-2,13
SLC24A6	7966600	NM_024959	12q24.13	solute carrier family 24 (sodium/potassium/calcium	-2,12

				exchanger), member 6	
RASGRP4	8036503	NM_170604	19q13.1	RAS guanyl releasing protein 4	-2,12
PLD3	8028791	NM_012268	19q13.2	phospholipase D family, member 3	-2,11
S100P	8093950	NM_005980	4p16	S100 calcium binding protein P	-2,09
GNG7	8032509	NM_052847	19p13.3	guanine nucleotide binding protein (G protein), gamma 7	-2,05
STK32B	8093858	NM_018401	4p16.2	serine/threonine kinase 32B	-2,03
ID1	8061564	NM_181353	20q11	inhibitor of DNA binding 1, dominant negative helix-loop-helix protein	-2,03
S100A9	7905571	NM_002965	1q21	S100 calcium binding protein A9	-2,02
PADI4	7898448	NM_012387	1p36.13	peptidyl arginine deiminase, type IV	-2,02
SIGLECP3	8030796	NR_002804	19q13.3	sialic acid binding Ig-like lectin, pseudogene 3	-2,01

Up-Regulated Genes

Gene Symbol	Probe Set ID	Gene Accession	Cytoband	Gene Description	log2 (R /WT)
CCL2	8006433	NM_002982	17q11.2-q12	chemokine (C-C motif) ligand 2	4,25
KYNU	8045539	NM_003937	2q22.2	kynureninase (L-kynurenine hydrolase)	4,14
NCALD	8152119	NM_001040624	8q22.2	neurocalcin delta	3,15
CD38	8094240	NM_001775	4p15	CD38 molecule	3,01
DUSP6	7965335	NM_001946	12q22-q23	dual specificity phosphatase 6	2,89
LOC643332	7973108	ENST00000258817	14q11.2	similar to Nonsecretory ribonuclease precursor (Ribonuclease US) (Eosinophil-derived neurotoxin) (RNase UpI-2) (Ribonuclease 2) (RNase 2)	2,86
ALOX5AP	7968344	NM_001629	13q12	arachidonate 5-lipoxygenase-activating protein	2,84
CIB2	7990674	NM_006383	15q24	calcium and integrin binding family member 2	2,82
GTSF1	7963817	NM_144594	12q13.13	gametocyte specific factor 1	2,67
SDC2	8147461	NM_002998	8q22-q23	syndecan 2	2,37
NEO1	7984704	NM_002499	15q22.3-q23	neogenin homolog 1 (chicken)	2,34
VCAN	8106743	NM_004385	5q14.3	versican	2,31
CXCL14	8114249	NM_004887	5q31	chemokine (C-X-C motif) ligand 14	2,31
CBR1	8068401	NM_001757	21q22.13	carbonyl reductase 1	2,27
METTL9	7993833	NM_016025	16p13-p12	methyltransferase like 9	2,24
GNG11	8134257	NM_004126	7q21	guanine nucleotide binding protein (G protein), gamma 11	2,16
PDGFA	8137670	NM_002607	7p22	platelet-derived growth factor alpha polypeptide	2,04
HLA-DRA	8178193	NM_019111	6p21.3	major histocompatibility complex, class II, DR alpha	2,03
